# A Report on the First Ex‐Utero Intrapartum Treatment (EXIT) Procedure in West Africa

**DOI:** 10.1002/ccr3.71397

**Published:** 2025-11-18

**Authors:** Betty Anane‐Fenin, Oluwayemisi Esther Ekor, Teresa Mensah, Victor Etwire, Joyce Ashong, Kenneth Kojo Baidoo, Grace N. A. Amoo‐Quaye, Joycelyn Akuorkor Ashong, Kofi Ulzen‐Appiah, Peter Appiah‐Thompson, Elizabeth Mercy Quartson, Hilda Danquah Konadu, Francis Koranteng, Vincent Kwesi Nuatror Nuavin‐Aseye, Isaac Kwakye, Nana Andoh Hanson, Nicholas Kumi

**Affiliations:** ^1^ Maternal‐Fetal Medicine Unit, Department of Obstetrics and Gynaecology Cape Coast Teaching Hospital Cape Coast Ghana; ^2^ Department of Anaesthesia and Pain Management, School of Medical Sciences University of Cape Coast Cape Coast Ghana; ^3^ Paediatric Surgery Unit, Department of Surgery Korle‐Bu Teaching Hospital Accra Ghana; ^4^ Paediatrics and Child Health Department Cape Coast Teaching Hospital Cape Coast Ghana; ^5^ Ear, Nose and Throat Department Korle‐Bu Teaching Hospital Accra Ghana; ^6^ Ear, Nose and Throat Department Cape Coast Teaching Hospital Cape Coast Ghana; ^7^ Maternal‐Fetal Medicine Unit, Department of Obstetrics and Gynaecology Komfo Anokye Teaching Hospital Kumasi Ghana; ^8^ Department of Pathology Cape Coast Teaching Hospital Cape Coast Ghana; ^9^ Department of Surgery, School of Medical Sciences University of Cape Coast Cape Coast Ghana; ^10^ Surgical Sub‐BMC Cape Coast Teaching Hospital Cape Coast Ghana; ^11^ Department of Anaesthesia and Critical Care Cape Coast Teaching Hospital Cape Coast Ghana; ^12^ Nicholas Kumi, Clinical Pharmacy and Drug Information Unit, Pharmacy Directorate Cape Coast Teaching Hospital Cape Coast Ghana

**Keywords:** cystic hygroma, EXIT procedure, ex utero intrapartum treatment, groundbreaking

## Abstract

The Ex utero Intrapartum Treatment (EXIT) is a complex perinatal intervention designed to secure the fetal airway while maintaining uteroplacental circulation during delivery. We report on the first application of this procedure in West Africa, describing our obstetric and resource‐constrained challenges in the management of a fetus with a large cervical cystic hygroma, and highlighting the complexities of EXIT outcomes. A report on the management of a large fetal anterior neck swelling with anticipated airway obstruction, presenting at 38 weeks 4 days has been presented. Through a multidisciplinary team collaboration, a clear working protocol and simulations, the first‐ever EXIT procedure in our subregion was performed, although an adverse neonatal outcome was encountered. The outcomes of EXIT procedures are unpredictable; hence, adequate counseling is required to embrace the outcome.

## Graphical abstract

The first ex‐utero intrapartum treatment (EXIT) procedure in West Africa. EXIT procedure can be done safely under resource‐constraints settings. Adverse perinatal outcomes can still occur, even after a successful EXIT procedure.

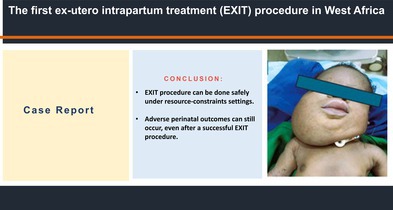

**Summary**

- EXIT procedures can be performed with strong multidisciplinary collaboration in resource‐constrained settings, and a successful intubation does not preclude the possibility of postnatal respiratory and circulatory failure.

## 1 Introduction

The Ex utero Intrapartum Treatment (EXIT) procedure is a specialized perinatal surgical intervention performed when airway obstruction is anticipated in a fetus; often due to large cervical masses such as cystic hygromas and teratomas, lung and mediastinal tumors, and congenital high airway obstruction syndrome (CHAOS). The primary goal of this high‐risk procedure is to establish a secure airway prior to umbilical cord clamping, ensuring placental support to prevent neonatal hypoxia. Unlike caesarean delivery, EXIT necessitates intentional uterine relaxation and partial fetal delivery to facilitate airway access under optimal conditions [1].

The EXIT procedure was initially developed for reversing tracheal occlusion in fetuses with severe congenital diaphragmatic hernia, but the procedure has since expanded for various treatment destinations: EXIT‐to‐airway, EXIT‐to‐resection, and EXIT‐to‐separation of conjoined twins [2]. The protocol for the technique, as described by Marwan et al. and Liechty et al., has served as the blueprint for EXIT over the years [1, 3]. In Africa, the procedure was first performed in Tygerberg Hospital in South Africa in December 2021 for a fetal mouth tumor [4]. It was followed shortly by Groote Schuur Hospital, where the procedure was performed for a fetus with congenital high airway obstruction syndrome (CHAOS) [5].

Despite the several successful EXIT outcomes reported, neonatal complications such as severe birth asphyxia and neonatal death can also occur, even after adequately securing the airway promptly [6]. Such cardiorespiratory dysfunction could be attributed to a failed fetal‐to‐neonatal cardiorespiratory transition. Cord compression, placental abruption, inadequate uterine relaxation, and cutting through the placental edge are examples of circumstances in which uteroplacental gas exchange could fail. Marwan and colleagues report on how polyhydramnios prevented the proper estimation of the placenta's proximity to the hysterotomy, leading to massive intrapartum hemorrhage and poor neonatal outcomes [1]. The presence of pathologies such as tracheomalacia, pulmonary hypoplasia, laryngeal atresia, and myometrial dysfunction has been identified as some of the underlying causes of severe post‐intubation respiratory distress. The large size of a cervical mass and tracheal deviation have also been reported to negatively affect the success of the procedure [7, 8].

The application of EXIT in Sub‐Saharan Africa is limited due to diagnostic challenges and inadequate logistics. This report describes the first documented EXIT procedure in West Africa, highlighting the complexities of managing a large fetal neck mass in resource‐limited and time‐constrained settings. It also emphasizes the importance of anticipating not only airway obstruction but also postnatal cardio‐respiratory instability, even after successful airway management.

## 2 Case Presentation

### 2.1 Case History and Examination

A 30 year‐old Gravida 2 Para 1 was referred to our maternal‐fetal medicine center at 38 weeks 4 days, after an ultrasound scan done 3 days prior to presentation revealed a large anterior neck mass that earlier obstetric scans had missed.

**FIGURE 1:**
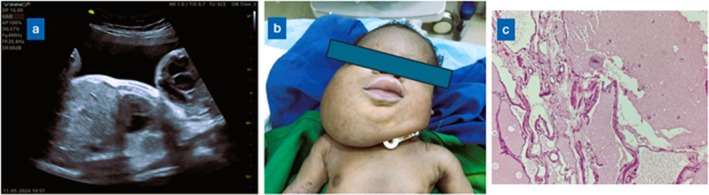
(a) Midsagittal view of the fetus showing the relationship between the mass and the chest. (b) Frontal view of the baby with the mass (c) Hematoxylin and Eosin (H&E) stain (X400) showing multiple varying‐sized thin‐walled cysts lined by flattened endothelial cells containing proteinaceous fluid and occasional foamy macrophages consistent with a lymphatic malformation (lymphangioma) 165 × 46mm (96 × 96 DPI).

On arrival at our center, a review of her records showed that she had no comorbidities, and the pregnancy had been uneventful. Her previous pregnancy, delivery, and puerperium were also normal. Physical examination findings were unremarkable. On ultrasound scan, a multicystic mass with areas of solid components and vascular flow was detected at the anterior neck, with predominance on the right. The mass measured about 11 cm below the mandible on the right and could not be differentiated from the surrounding tissue. There were also areas of vascular flow noted within the solid components of the mass. Limited anatomy scan did not reveal any other structural abnormalities or features of chromosomal or genetic anomalies, and the fetal presentation was cephalic. The two differential diagnoses considered were cystic hygroma and cervical teratoma (Figures 1a and Video 1).

**VIDEO 1:** The mass is shown in the midsagittal plane (This video has no sound). Video content can be viewed at https://onlinelibrary.wiley.com/doi/10.1002/ccr3.71397.

### 2.2 Work‐Up and Differential Diagnoses

Although ultrasound is the first‐line investigation for a fetal neck mass, magnetic resonance imaging (MRI) and computed tomography (CT) scans can provide a more detailed view of the surrounding structures, which helps in assessing the extent of the mass and its potential impact on the airway. CT scan is, however, less preferred due to radiation exposure to the fetus, and its limited ability to assess soft tissue structures and to differentiate between cystic and solid tissue. A definite diagnosis, however, is through tissue histopathology.

MRI was not available to us at the time, and a CT scan could also not be performed as a second option, due to financial constraints. The suspicion of a cystic hygroma, with cervical teratoma as a differential diagnosis in this report, was based solely on the sonographic features described above. Histopathology later confirmed a cystic hygroma.

Differential diagnoses of cystic hygroma (lymphangioma) include fetal goiter, thyroglossal cyst, branchial cleft cyst, hemangioma, neuroblastoma, dermoid cyst, cervical teratoma, and the much rarer cervical sarcoma. A fetal goiter was unlikely because the mass was neither homogenous nor symmetrical. Similarly, a thyroglossal cyst was unlikely because it was not a midline mass. A branchial cyst was ruled out because the mass was neither echogenic nor unilateral. A hemangioma was also ruled out, because the mass neither appeared solid nor had abundant venous and low‐resistance arterial waveforms on color Doppler. Contrary to a lymphangioma, a neuroblastoma appears as a hyperechoic heterogeneous mass (mainly solid but with few cystic components) with increased internal vascularity.

Invasive testing for karyotyping and microarray, which are ideal to rule out chromosomal and genetic anomalies, could not be done due to financial constraints. Despite the unavailability of MRI and CT‐scan assessments, airway obstruction was strongly anticipated due to the extent of the mass on ultrasound.

### 2.3 Management and Outcome

The patient was counseled on the fetal condition and the need for an EXIT procedure, which she consented to. She was admitted for maternal and fetal surveillance because she was at risk of going into labor before the procedure. Her preoperative laboratory investigations were unremarkable. Blood grouping and crossmatch were done against 3 units of whole blood, in anticipation of postpartum hemorrhage due to her history and the uterine hypotonia to be induced.

**FIGURE 2:**
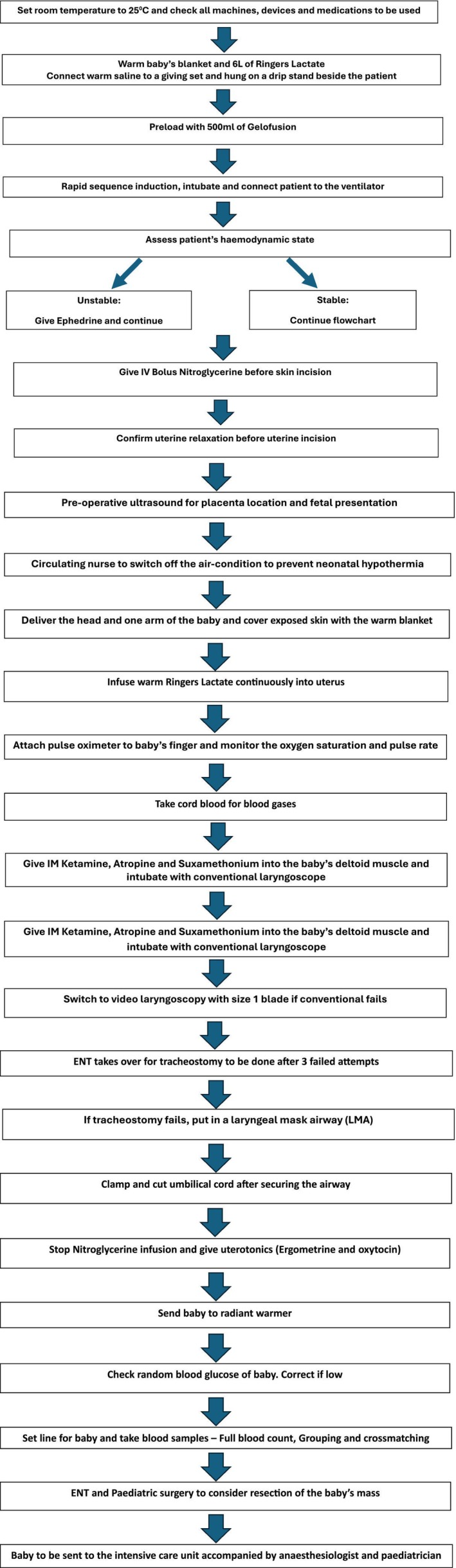
Flowchart for the EXIT procedure.

A multidisciplinary team was immediately convened, comprising maternal‐fetal medicine specialists, an anesthesiologist, a pediatrician, otorhinolaryngologists, a pediatric surgeon, and perioperative nurses. The blood bank was also informed. An EXIT procedure was scheduled for the 15th of May 2024, 6 days after the diagnosis. Before that, one online meeting and a physical simulation exercise were held. A workflow chart was drawn to show the entry point for each specialty (Figure 2). The chart was printed in bold fonts and pasted at vantage points in the operating room for easy access to team members.

About 45 min before the surgery, uterine contractions of 2 in 10 lasting 20 s were observed in the patient. Tocolysis with inhalational salbutamol and IV Magnesium sulfate was initiated because they were readily available agents. Her vital signs were normal, and cardiotocography was reactive. Immediately before the induction of anesthesia, spontaneous rupture of the chorioamniotic membranes occurred. The liquor was clear and non‐offensive, and cord prolapse was ruled out. The fetal heart rate before the induction of anesthesia was 152 beats per minute and regular, and the amniotic fluid index was 6 cm.

Under general anesthesia and uterine relaxation in the operating room, a low transverse uterine incision was made to partially deliver the fetal head to the shoulders. The neck mass was noted to be soft and measured 23 cm and 12 cm in the horizontal and vertical planes, respectively (Figure 1b). There were no obvious features suggestive of Noonan's syndrome, Turner's syndrome or Down syndrome. The initial neonatal vital signs were stable (oxygen saturation 95%; heart rate 91 beats per minute, which improved shortly to 103 beats per minute; random blood glucose 6.4 mmol/L; temperature 36.7°C). However, oxygen desaturation was noted soon afterwards (SPO2 of 41%). Intubation was achieved within 5 min of delivery, and oxygen saturation improved immediately. The cord was cut and ligated, and the baby was put under the radiant warmer, but the oxygen saturation began to deteriorate again, so a tracheostomy was performed as a second option. However, severe bradycardia and several cardiac arrests followed, leading to the death of the neonate despite 2 h of rigorous resuscitation.

The placenta was delivered by controlled cord traction, and there were no retroplacental clots. No active bleeding from the uterus occurred, even without uterine staples. Uterine contraction was adequate throughout the repair.

A histopathological assessment of the mass confirmed cystic hygroma (Figure 1c). There was a deviation of the trachea to the left, but the rest of the respiratory and cardiovascular systems were structurally normal.

## 3 Discussion

This is a report on the first EXIT procedure in the West African sub‐region, highlighting the strength in multidisciplinary teamwork and the challenges encountered in the management of a huge fetal cystic hygroma.

Cystic hygroma is a congenital malformation of the lymphatic system in the neck. It is the most common form of lymphangioma, with 75% located on the neck, 20% in the axillary area, and about 5% in the mediastinum, abdominal cavity, and the extremities [9, 10]. Chromosomal abnormalities such as Turner's syndrome and Trisomies 21 and 18 are associated in 50% of cases, while genetic conditions such as Noonan syndrome, multiple pterygium syndrome, Fryns syndrome, and Neu‐Laxova syndrome are found in about 40% [11, 12]. Fetal airway obstruction can occur due to extrinsic compression from mass effect, or intrinsic obstruction due to reasons such as laryngeal atresia.

Ultrasound is the first line for the diagnosis of cystic hygroma. The sonographic feature in the first trimester is a cystic structure located in the occipital‐cervical region of the fetal neck with a nuchal ligament or midline septum [13]. In the advanced stages, it typically manifests as a complex tumor in the anterior or antero‐lateral neck, which is predominantly multicystic with thin walls, with solid areas in some cases. The solid components can be explained by the presence of either areas of lymphatic obstruction in between fibrous tissue and muscle, or remnants of abnormal undilated lymphatics that have lumped together [14]. The mass is also asymmetric, with poor differentiation from the surrounding tissue [15].

Differential diagnoses of cystic hygroma include congenital goiter, branchial cleft cyst, neuroblastoma, hemangioma, and cervical teratoma [10, 15, 16, 17, 18, 19, 20]. In our case, sonography revealed a predominantly multicystic mass with some solid components, which had positive vascular flow on Doppler. Fetal MRI and CT scans could not be done due to the already stated reasons.

Obstetric complications such as preterm premature rupture of membranes, onset of labour, development of fetal hydrops, or maternal mirror syndrome could have negative implications on the procedure. In their report, Lin et al. revealed that 32% of procedures were performed before the scheduled time due to some of these reasons [21]. Similarly, Zaretsky and colleagues describe a challenging procedure in the setting of preterm labour, cord prolapse, and sub‐optimal uterine relaxation [22]. In our report, we also cite the occurrence of spontaneous labour and rupture of membranes a few minutes before the scheduled surgery time.

There are several cases of a successful EXIT, but it is worth mentioning that adverse neonatal outcomes may occur when intubation fails or even after securing the airway [6]. Technically successful intubation, therefore, does not guarantee survival. Liechty et al. recount the death of a neonate due to severe respiratory distress following a tracheostomy, in which autopsy revealed pulmonary hypoplasia as a result of the lungs being wedged in the apexes of the chest [3]. Similarly, Lin and colleagues report on 65 EXIT procedures with an overall fetal/neonatal mortality rate of 15% even after securing the airway [21]. There was one intraoperative fetal demise, which was later found to be due to a rudimentary trachea superior to the carina, in a case of congenital high airway obstruction. Four neonates with huge cervical teratomas who required complex airway management, such as tracheostomy and neck dissection, still died on account of pulmonary hypoplasia. Again, a third of neonates in another review died due to airway management failure or complications of an underlying respiratory disorder [6]. These underlying conditions cause severe hypoxia and fetal acidosis, which can lead to metabolic derangement and neonatal death [22].

In our report, apart from the presence of tracheal deviation, there were no structural airway or cardiac pathologies noted at autopsy. We speculate that the fatal hypoxia our neonate encountered was due to an idiopathic dysfunctional transition from the fetal to the neonatal circulation.

## 4 Conclusion

Our case report is one of the few EXIT cases with an adverse neonatal outcome despite prompt and adequate airway management. We advise that in the context of a lack of detailed airway assessment in utero for a large neck mass, an obstruction should be anticipated. Patients must also be counseled that aside from the expectation of difficult airway management, cardiorespiratory collapse after successfully securing the airway is not impossible.

## Author Contributions

**Betty Anane‐Fenin:** conceptualization, supervision, validation, writing – original draft. **Oluwayemisi Esther Ekor:** writing – original draft, writing – review and editing. **Teresa Mensah:** writing – review and editing. **Victor Etwire:** writing – review and editing. **Joyce Ashong:** validation, writing – review and editing. **Kenneth Kojo Baidoo:** writing – review and editing. **Grace N. A. Amoo‐Quaye:** writing – original draft, writing – review and editing. **Joycelyn Akuorkor Ashong:** writing – review and editing. **Kofi Ulzen‐Appiah:** writing – review and editing. **Peter Appiah‐Thompson:** writing – review and editing. **Elizabeth Mercy Quartson:** writing – review and editing. **Hilda Danquah Konadu:** writing – review and editing. **Vincent Kwesi Nuatror Nuavin‐Aseye:** writing – review and editing. **Francis Koranteng:** writing – review and editing. **Isaac Kwakye:** writing – review and editing. **Nana Andoh Hanson:** writing – review and editing. **Nicholas Kumi:** writing – review and editing.

## Ethics Statement

Ethical approval was given by the Cape Coast Teaching Hospital Management and Ethical Review Committee.

## Consent

Written informed consent was obtained from the patient for publication of this case report and the accompanying images in accordance with the journal's patient consent policy.

## Conflicts of Interest

The authors declare no conflicts of interest.

## Data Availability

Data supporting the conclusions of this report are contained within the report. Additional non‐relevant patient data are protected under patient privacy regulations and policies.
